# Assessment of the Incidence of Enteric Adenovirus Species and Serotypes in Surface Waters in the Eastern Cape Province of South Africa: Tyume River as a Case Study

**DOI:** 10.1100/2012/949216

**Published:** 2012-11-22

**Authors:** Timothy Sibanda, Anthony I. Okoh

**Affiliations:** Applied and Environmental Microbiology Research Group (AEMREG), Department of Biochemistry and Microbiology, University of Fort Hare, Alice 5700, South Africa

## Abstract

TaqMan real-time PCR was used for the detection and quantitation of adenoviruses in Tyume River water samples over a 12-month period. A total of 72 samples were analysed, and 22 samples were positive for adenovirus. Of the positive samples, 18 were collected from downstream sampling points. Among the downstream sampling points, adenovirus detection rate increased with distance downstream, being 28%, 33%, and 39% for Alice, Drayini, and Manqulweni, respectively. The Alice sampling site had the highest concentrations of adenovirus ranging between 6.54 × 103 genome copies/L and 8.49 × 104 genome copies/L. The observed trend could have been expected considering the level of anthropogenic activities in areas along the lower stretch of Tyume River, with the major one being the effluent of treated and semi treated sewage from wastewater treatment facilities. Adenovirus detection was sporadic at most sampling sites. Multiplex conventional PCR was used for the detection of clinically important adenovirus species B, C, and F and their serotypes. Species C and F adenoviruses were detected in 77% and 18% of the samples, respectively. Most adenovirus positive samples were obtained from areas of increased population densities. The presence of adenoviruses may confirm the risk of its transmission to the human population.

## 1. Introduction

Enteric viruses are present in high concentrations in faeces of infected persons [1, 2]. In areas lacking adequate sanitary infrastructure these viruses eventually find their way into the environment with minimal or no reduction in their numbers and/or infectivity. Adenovirus is the most prevalent of enteric viruses in water environments worldwide [3]. It is also the only DNA viral pathogen in the enteric virus family and hence tends to outlast other enteric viruses in environmental waters as a result of its thermostability [4]. Human adenoviruses (HAdV) are a major cause of clinical infections including gastroenteritis, conjunctivitis, and respiratory diseases [5] and are the second most important viral pathogens of infantile gastroenteritis after rotavirus [1, 6]. Adenoviruses are members of the Adenoviridae family and include 70 nm to 100 nm nonenveloped icosahedral viruses. At present, there are 51 serotypes of adenoviruses, about 30% of which are pathogenic in humans, most causing upper respiratory tract infections. The serotypes are classified into six species, designated species A to F [7, 8]. The risk posed by adenovirus species F serotypes 40 and 41 as leading causes of childhood diarrhea led the Environmental Protection Agency (EPA) to enact the Information Collection Rule in 1996, which required all water utilities serving more than 100,000 households to monitor their source water for viruses [9]. Serotypes of species A, B, and C have been, to a lesser extent, linked to acute gastroenteritis in infants [10] with species B and C adenoviruses linked to outbreaks of pharyngoconjunctivitis in recreational waters [11]. Adenovirus infections have been observed to occur throughout the year with little or no seasonal variation in shedding [12]. 

The role water plays in the epidemiology of HAdV, as well as the potential health risks constituted by these viruses in water environments, is widely recognised [13, 14]. While faecal contamination of the water environment is currently being monitored only with bacterial indicators, it is vital to point out that bacterial and viral contaminations are not necessarily associated and linked with each other [15, 16]. Monitoring specific virus pathogens and their relative numbers in water samples would provide more reliable information for risk assessments of waterborne viral infections [17]. The presence of enteric viruses in sewage and hence in environmental surface waters reflects the infectious status of the population [18] and constitutes a public health risk [19]. Despite large advances in water and wastewater treatment, waterborne diseases still pose a major worldwide threat to public health [17], more so in developing countries where a substantive portion of the human population still rely on untreated surface waters for domestic purposes. Infectious adenoviruses have previously been detected in high frequencies in surface waters used for drinking water supplies in South Korea and South Africa [20], and elsewhere [21, 22]. Inadequate chlorination during conventional drinking water treatment may fail to remove all viral pathogens, especially adenoviruses (owing to their increased resistance compared to other enteric viruses), from water, more so when the source water is heavily polluted. 

The sparsely populated upstream and the presence of a hospital, town, and university midstream of Tyume River catchment make it an interesting study site. The student population at the University of Fort Hare comprises people from different geographic regions and the wastewater effluent discharged from the university's wastewater treatment plant is likely to be contaminated with a range of viral pathogens. Effluent from Victoria Hospital is equally likely to contain a variety of pathogens as well. Effluents from the University of Fort Hare, Alice Town, and Victoria Hospital wastewater treatment plants are discharged directly into Tyume River. So far, no studies have indicated the occurrence of adenoviruses in surface waters in the Eastern Cape Province of South Africa. Therefore, the purpose of this study was to use real-time PCR for the detection and quantitation of adenoviruses in Tyume River since it serves as a public water supply to the Nkonkobe Local Municipality. Because low levels of adenoviruses in drinking water could result in significant risks of infection and mortality in sensitive subpopulations [23], the presence of adenoviruses may confirm the risk of its transmission to the human population.

## 2. Methods and Materials

### 2.1. Sampling

One litre water samples were collected once monthly for 12 months (August 2010 to July 2011) from six sampling sites along Tyume River. Samples were transported in cooler boxes to the Applied and Environmental Microbiology Research Group (AEMREG) Laboratory at the University of Fort Hare, Alice, for processing and analyses. Processing of samples was done within 6 h of sample collection. In all, a total of 12 samples per site were collected giving a total of 72 samples for the 12-month sampling period. 

### 2.2. Study Site

**Figure 1:**
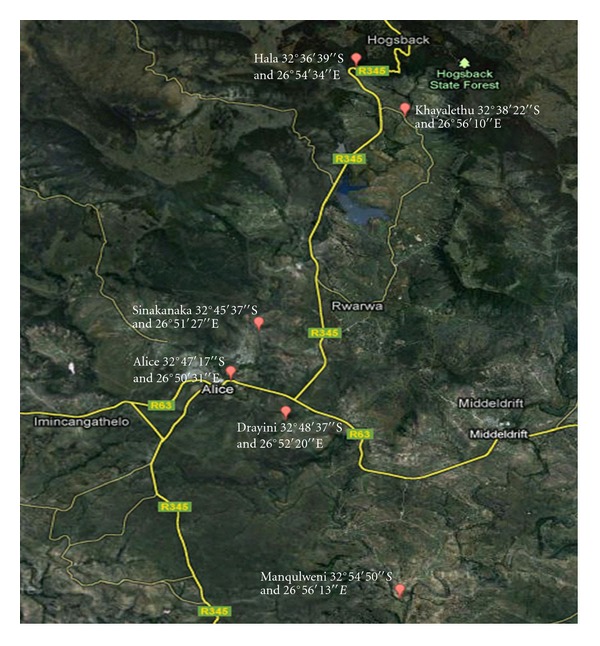
Map showing the sampling sites along Tyume River.

The Tyume River is located in the Nkonkobe local municipality, under the Amathole District Municipality, in the Eastern Cape Province, South Africa. It flows from the upper part of the Amathole Mountains in Hogsback, passing through the lower coastal escarpment down to Alice through several rural settlements and finally joins the Keiskamma River at Manqulweni community. Close proximity of the river to its host communities makes it ideal for utilisation for domestic activities where piped potable water is not available. The Tyume River also feeds the Binfield Park Dam which serves as source of raw water for several water treatment plants in the area where water is treated and reticulated to Alice Town and surrounding rural settlements. The sampling sites for the Tyume River catchments include Hala, Khayalethu, Sinakanaka, Alice, and Drayini and Manqulweni communities.  Figure 1 below is a map showing the sampling sites along Tyume River.

### 2.3. Concentration of Viruses in Water Samples

Viruses in water samples were concentrated following the adsorption-elution method as described by Haramoto et al. [24], with some modifications. This method showed recovery yields of 56%  ±  32% (*n* = 37) for surface-water samples inoculated with polioviruses, and it is based on electrostatic interactions. Under neutral pH conditions viruses are negatively charged and are positively charged under acidic conditions. Multivalent cations (Mg^2+^, Al^3+^) can change the surface charge of viruses thereby allowing adsorption to negatively charged membranes. An aliquot of 5 mL of 250 mM AlCl_3_ was passed through an HA filter (0.45 *μ*m pore size and 47 mm diameter, Millipore) attached to a glass-filter holder, to form a cation (Al^3+^-) coated filter. Subsequently, 1 L of the water sample was passed through the filter. A volume of 200 mL of 0.5 mM H_2_SO_4_ was then passed through the membrane and viral particles were eluted with 10 mL of 1 mM NaOH. Eluates were carefully placed in a tube containing 0.1 mL of 50 Mm H_2_SO_4_ and 0.1 mL of 100× Tris-EDTA (TE) buffer for neutralisation before further concentration. The concentrate was subjected to further concentration using Centriprep YM-50 ultrafiltration device (Millipore) to obtain a final volume of approximately 700 *μ*L. The concentrates were stored at −80°C until ready for use. Storage of viruses at temperatures of below −60°C has been shown to result in insignificant loss of both titre and infectivity for periods longer than a decade [25, 26].

### 2.4. Extraction of Adenovirus DNA

Two sample aliquots (200 *μ*L each) of concentrated virus samples were prepared, one set of which was spiked with the specific virus control for quality assurance. Both sets were used for the extraction of viral DNA and purification with commercially available kits (Quick-gDNA MiniPrep; Zymo Research, USA) following the manufacturer's protocol. Purified viral DNA was eluted in 60 *μ*L of DNase-free water. 

### 2.5. Quantification of Adenovirus Genome by Real-Time PCR Assay

The concentrations of human adenovirus in the river water samples were estimated by using quantitative PCR (qPCR) with a TaqMan probe. Quantitative detection was performed using a StepOnePlus PCR System (OPTIPLEX 755, Applied Biosystems), forward primer JTVX(F) 5′-GGACGCCTCGGAGTACCTGAG-3′, reverse primer JTVX(R) 5′-ACIGTGGGGTTTCTGAACTTGTT-3′, and TaqMan probe JTVX(P) 5′-FAM-CTGGTGCAGTTCGCCCGTGCCA-MGBFQ-3′ [1, 6, 29]. (FAM, 6-carboxyfluorescein (reporter dye); MGBNFQ, minor groove binder/nonfluorescent).

Quantification of AdV by qPCR was done following a one-step reaction in a 96-well plate. The wells were loaded with 20 *μ*L of a reaction buffer (containing 12.5 *μ*L of 2× TaqMan universal PCR MasterMix Applied Biosystems, 400 nM sense primer, 400 nM antisense primer, and 250 nM TaqMan probe and PCR grade water [30]). Subsequently, 5 *μ*L aliquots of sample DNA were added with mixing to give 25 *μ*L total reaction mixtures. The plate was sealed and loaded into the thermocycler under the following cycling conditions; 15 min at 95°C for *Taq* activation, followed by 45 cycles of denaturation at 95°C for 10 s, annealing at 55°C for 30 s, and extension at 72°C for 20 s. Fluorescence data was collected at the end of each cycle. The primers were tested for cross reactivity by substituting our target DNA with nontarget DNA but no cross reactivity was observed. 

The standard curve was formulated as described by Haramoto et al. [30]. Briefly, DNA was extracted from an adenovirus ATCC positive strain (ATCC VR-930) using commercially available extraction kits. The DNA was then quantified using a Qubit fluorometer (http://www.invitrogen.com/site/us/en/home/brands/Product-Brand/Qubit.html) and diluted by serial tenfold dilution. The sample extracts and standards' samples were subjected to real-time PCR simultaneously, followed by analysis using SDS software (Applied Biosystems) to obtain quantitative data on the titre of viral DNA in a well. Two wells each were used for the standard, negative control (no template control) and sample, and the average used for subsequent calculations. The total number of viruses in the viral suspensions and eluted samples was estimated by multiplying the titre of viruses per millilitre by the volumes of the samples. By using control viral samples, sensitivity of detection was demonstrated to be fewer than 10 copies of viral genome per reaction and quantitative linearity was demonstrated to be from 10 to 10^6^ copies of input viral DNA.

### 2.6. Detection of Adenovirus Species and Serotypes

Serotype-specific multiplex PCR assays as described by Metzgar et al. [27] were used to detect the epidemiologically important serotypes, Ad3, Ad7, and Ad21 (belonging to species B), Ad1, Ad2, Ad5, and Ad6 (belonging to species C), and Ad4 (belonging to species E). The primers used are shown in Table 1 below. The F species serotypes Ad40 and Ad41 were detected using serotype-specific primers K402 and K403. AdV serotypes 40 and 41 were separated by digesting the PCR product with restriction enzyme ACC1 which cannot digest the AdV41 PCR product but restricts the AdV40 PCR product to band size of approximately 94 bp and 58 bp while the AdV41 product remains 152 bp. For quality assurances, the specific virus strains were used as controls.

### 2.7. Controls

Each test included two controls; a positive control consisting of a spiked sample containing predetermined concentrations of viral DNA (standard) and a negative control consisting of PCR-grade water and MasterMix formulation. The entire control virus strains (Table 2) used were obtained from ATCC and preserved at −80°C. 

### 2.8. Statistical Analysis

Analyses were made using the Statistical Package for the Social Sciences (IBM SPSS Statistics release 19; IBM, USA). One-way ANOVA and Tukey's Studentized Range (HSD) Test were used to test differences among all possible pairs of treatments while Pearson's correlation coefficient and Spearman's rank correlation test were used for correlation studies. 

## 3. Results

**Figure 2:**
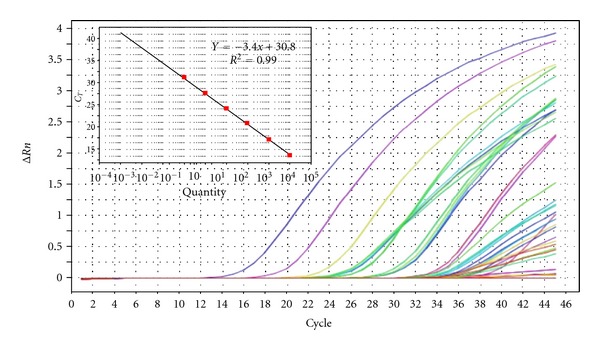
Amplification plot for adenovirus quantitation in Tyume River.

**Figure 3:**
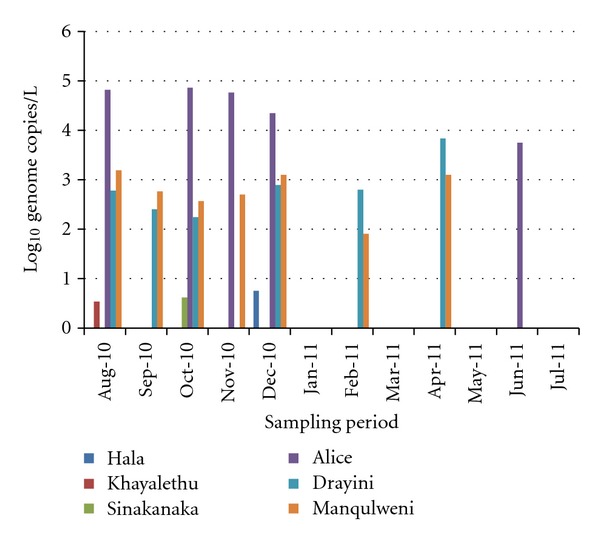
Log_10_ genome copies/L of adenovirus at selected sites along Tyume River.

The concentrations of adenovirus detected in this study ranged between 1.0 × 10^0^ genome copies/L and 8.49 × 10^4^ genome copies/L. Of the 72 samples collected over a 1-year period, 22 samples were positive for adenovirus giving a detection rate of 31%. Of these, 82% (18/22) were collected from downstream sampling sites (Alice, Drayini, and Manqulweni). Statistical analysis showed that adenovirus detection was significantly higher among the downstream sampling sites (*P* < 0.05) compared to the upstream sites (Hala, Khayalethu, and Sinakanaka).  Figure 2 shows the amplification plot (with standard curve) while Figure 3 shows the log_10_ genome copies/L obtained after real-time PCR assay for adenovirus.

**Figure 4:**
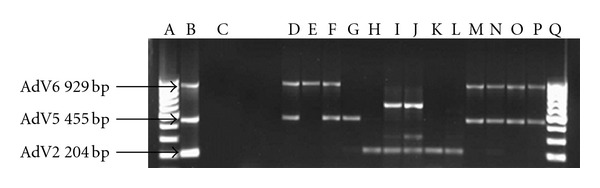
EtBr-stained agarose gel picture showing HAdV species C serotypes 2, 5, and 6. Lanes A and R = DNA Ladder; Lane B = positive control; Lane C = negative control; Lane E = Alice (Dec 2010); Lane F = Alice (Nov 2010); Lane G = Alice (Oct 2010); Lane H = Alice (Aug 2010); Lane I = Manqulweni (Dec 2010); Lane J = Manqulweni (Oct 2010); Lane K = Manqulweni (Sept 2010); Lane L = Manqulweni (Aug 2010); Lane M = Manqulweni (March 2011); Lane N = Drayini (Dec 2010); Lane O = Drayini (Sept 2010); Lane P = Drayini (Aug 2010); Lane Q = Drayini (Feb 2011).

**Figure 5:**
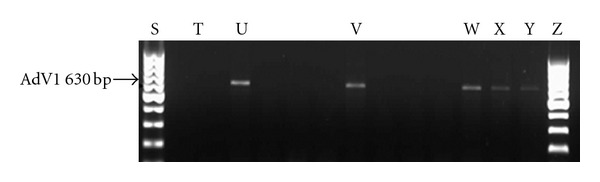
EtBr-stained agarose gel picture showing HAdV species C serotype 1. Lane S = DNA ladder; Lane T = negative control; Lane U = positive control; Lane V = Manqulweni (Oct 2010); Lane X = Drayini (Dec 2010); Lane Y = Drayini (Sept 2010); Lane Z = Drayini (Feb 2011).

**Figure 6:**
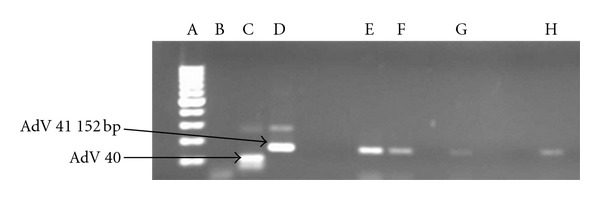
EtBr-stained agarose gel picture showing HAdV species F serotypes 40 and 41. Lane A = DNA Ladder; Lane B = negative control; Lane C = positive control (HAdV 40); Lane D = positive control (HAdV 41); Lane E = Alice (Oct 2010); Lane F = Alice (Aug 2010); Lane G = Drayini (Dec 2010); Lane H = Manqulweni (Sept 2010).

The highest concentrations of adenovirus ranging between 6.54 × 10^3^ genome copies/L and 8.49 × 10^4^ genome copies/L were recorded in samples collected from the Alice sampling site between August 2010 and June 2011. Of the 22 samples which were positive for adenovirus by real-time PCR, 17 were positive for species C adenovirus and of this, 6 were positive for both adenovirus serotypes 6 and 7, 1 sample was positive for each of serotypes 6 and 7, and 5 were positive for adenovirus serotype 2 (Figure 4), while 4 were positive for adenovirus serotype 1 (Figure 5). None of the samples were positive for adenovirus species B or A while 4 of the samples were positive for adenovirus species F serotype 41 (Figure 6).

## 4. Discussion

The downstream stretch of Tyume River flows through areas of high population density characterised by the presence of the small town of Alice, the University of Fort Hare, and Victoria Hospital whose combined population is approximately 48,000 [31]. Compared with the downstream Tyume, the extreme upper reaches of Tyume River are sparsely inhabited with a rural settlement setting. The higher prevalence of adenovirus in the downstream stretch of the river could therefore be explained in terms of increased human pressure on the environment. The most probable of the anthropogenic activities contributing to contamination of natural water sources with enteric pathogens could be the discharge of partially/untreated wastewater effluents from domestic and municipal sewage into the river. A study done by La Rosa et al. [32] on wastewater effluents found not only elevated concentrations of enteric viruses, among them adenovirus, but also found infective virions in the final effluents. The primers used for the TaqMan real-time PCR assays in this study were specific for human adenoviruses and had previously been used to detect human adenoviruses in environmental samples [6]. Results obtained in this study seem to consolidate this fact since the detection rate was heavily skewed to the downstream of the river where human influence is greater. This is despite the fact that the upstream Tyume is exposed to other potential sources of pollution such as livestock watering which happens directly in the river channel.

The highest concentrations of adenovirus were found at the Alice sampling site which lies immediately downstream from sewage outfall points from Victoria Hospital and the northern suburbs of Alice Town which include Ntselamantsi and Lower and Upper Gqumashe. The discharge of hospital wastewater effluents into the river could be a major source of enteric pathogens owing to the presence at the hospital of patients suffering from a wide range of ailments, viral gastroenteritis a possibility among them. Because human adenoviruses (HAdVs) are double-stranded DNA viruses, they have remarkable stability with regards to several physical conditions such as pH, temperature, and moisture. In addition, their resistance to commercially available disinfectants or wastewater treatments contributes significantly to their persistence in the environment [33–36].

Among the downstream sampling points, adenovirus detection rate increased with distance downstream, being 28%, 33%, and 39% for Alice, Drayini, and Manqulweni, respectively. This trend has been noted in previous studies and attributed to the fact that HAdVs are likely able to survive in effluents of wastewater treatment plants discharged upstream in the river, survive sunlight inactivation, and be transported to the downstream areas [29]. Also, the increased detection especially at Drayini compared to Alice could be attributed to effluent discharge from the University of Fort Hare wastewater treatment plant which discharges just upstream of the Drayini sampling site. The university comprises a large number of people in a relatively small piece of land, many of them coming from different geographical regions of the African continent. There is therefore a possibility of a range of enteric pathogens (including adenoviruses) being prevalent in the sewage of such an institution.

One-way ANOVA analyses of results showed that adenovirus detection did not differ by season in most sampling points except at Drayini and Manqulweni where its detection significantly differed between winter and spring (*P* < 0.05). This suggests that adenovirus was an all-season contaminant of the river, in agreement with previous findings [1]. Its sporadic detection may also probably have been encouraged by the temperature of the river which was generally within the range of optimal virus survival (<23°C) throughout the year (data not shown), in agreement with previous findings of Lipp et al. [37]. Also, there was no direct relationship between adenovirus detection in this study and rainfall events suggesting that the pollution of the river from human sources may be sporadic throughout the year, which is independent of rain events, as has been reported elsewhere, Choi and Jiang [38]. 

### 4.1. Adenovirus Characterisation

Real-time PCR positive samples for HAdV were further subjected to multiplex conventional PCR for detection of clinically important adenovirus species B, C, and F and their serotypes. The HAdV serotypes 40 and 41 have long been recognised as the main etiological agents of 1–20% of acute viral gastroenteritis in children [6, 39–41]. While Shimizu et al. [41] state that 50% of all adenoviruses found in stool specimens are types 40 and 41 (species F); there were more samples positive for AdV C than F in this study. This result was unexpected but could as well serve to indicate the most common AdV species in circulation in the human population in Tyume River catchment at the time. Of the AdV F positive samples, 100% were AdV 41 (Figure 6: lane E–H). This corroborates the findings of other researchers [42, 43] who also found that AdV 41 is more prevalent than AdV 40, a result other researchers attributed to a decline in AdV 40 infection and elevation of infection with AdV 41 [44, 45]. AdV types 40 and 41 can cause mortalities as much as 50% in immunocompromised individuals [45]. Considering that these viruses are shed for extended periods in faeces, urine, and respiratory secretions of infected persons [46], their low prevalence in this study suggests a low incidence of species F infections in the host population. 

Species B and C adenoviruses have been linked to outbreaks of pharyngoconjunctivitis [11] and may play an important role in the transmission of respiratory diseases in recreational waters through aerosol transmission [47]. AdC serotypes are also associated with a wide variety of illnesses in immunocompromised patients and, on rare occasions, in healthy adults [27]. Type 2 adenoviruses are generally associated with pneumonia and childhood respiratory diseases [48]. AdV serotypes 1, 2, and 5 are implicated in 5–10% of childhood respiratory diseases, which are, however, mostly self-limiting [20]. These viruses also cause conjunctivitis in healthy adults [20]. The unusual prevalence of AdV C serotypes I in this study could be explained by the fact that after acute infection, AdV types 1, 2, and 5 may be shed in stool for months to years [20], which probably causes the endemic spread to other susceptible groups largely through ingestion of contaminated water. Gray et al. [49] found that multiple AdV serotype infections are more frequent in immunocompromised patients (30%) than in immunocompetent patients (5%). Abe et al. [50] also found that the persistence of AdV C infections is higher for younger age groups, in which primary infections are predominantly caused by species C. The presence of these HAdV serotypes in the river therefore suggests that a significant portion of the human population in this catchment could have suffered from AdV-induced illness especially between August and December 2010 which is the period with 85% of all adenovirus detections. Since enteric viruses, of which HAdV is one, are present in the faeces of infected patients in high concentrations [7, 30], the decline in the detection rate of HAdV in the year 2011 may also be an indication of the declining incidence of HAdV infections among the human population living in the Tyume River catchment. Adenovirus detection in this study seemed to be strongly associated with point-source human faecal pollution, an observation that agrees with previous findings [51]. 

Monitoring of river water for enteric viruses could be one of the suitable approaches to understand the actual prevalence of viruses in the river catchment area, because most urban rivers receive effluents from multiple wastewater treatment plants that contain viruses shed from all patients in the catchment area [52]. In this study, adenovirus was detected at all the six sampling sites along the Tyume River howbeit in varying frequencies and titres. The results obtained seem to correlate adenovirus occurrence in river water to increased population densities in river catchments. Potential risk of infection from waterborne adenovirus infections may therefore be higher in downstream as compared to upstream stretches of the river, especially where untreated river water is used for drinking and recreational purposes. Previous studies done elsewhere also demonstrated increased prevalence of enteric viruses in downstream stretches of rivers compared to the upstream stretches [52]. Areas with high population densities also seem to shed more species of adenovirus into the environment as evidenced by the detection of AdV 1, AdV 2, AdV 5, AdV 6, and AdV 41 at Alice and Drayini sampling sites which are located in areas commanding higher population densities than at any other sites along Tyume River. 

### 4.2. Conclusions

It should be noted that this is the first report of HAdV detection in Eastern Cape environmental waters using qPCR methods to the best of our knowledge. This study and numerous other studies of its nature have demonstrated that real-time PCR is a powerful tool for rapid determination of enteric viruses in environmental samples and represents a considerable advancement in pathogen quantification in aquatic environments. The high prevalence of HAdV in Tyume River waters obtained in this study indicates an elevated public health risk especially among the young and the immunocompromised individuals, who consume, swim, or bath in these waters given that a significant proportion of the rural populace in this province still rely on untreated surface water sources for their domestic water needs. Also, farmers, farm workers, and consumers of fresh farm produce from farms irrigated with Tyume River water may be at risk of infection from adenoviruses. Farmers and farm workers could be exposed to AdV infections through aerosols from sprinkler irrigation schemes such as the ones around Alice while consumers could be exposed to infection from eating raw vegetables which are either inadequately washed or are washed using the same contaminated river water. The findings of this research therefore highlight the lurking dangers of using contaminated surface water and the need for routine monitoring of such waters for protection of public health.

## Figures and Tables

**Table 1:** Primers for detection of adenovirus serotypes.

Species	Serotype	Primer	Sequence (5′ to 3′)	Target region
B	Ad3	Ad3F	GGTAGAGATGCTGTTGCAGGA	Ad3 hexon
		Ad3R	CCCATCCATTAGTGTCATCGGT	
	Ad7	Ad7F	GGAAAGACATTACTGCAGACA	Ad7 hexon
		Ad7R	AATTTCAGGCGAAAAAGCGTCA	
	Ad21	Ad21F	GAAATTACAGACGGCGAAGCC	Ad21 hexon
		Ad21R	AACCTGCTGGTTTTGCGGTTG	
C		AdCF	TGCTTGCGCTHAAAATGGGCA	AdC fibre
	Ad1	Ad1R	CGAGTATAAGACGCCTATTTACA	Ad1 fibre
	Ad2	Ad2R	CGCTAAGAGCGCCGCTAGTA	Ad2 fibre
	Ad5	Ad5R	ATGCAAAGGAGCCCCGTAC	Ad5 fibre
	Ad6	Ad6R	CTTGCAGTCTTTATCTGAAGCA	Ad6 fibre
E	Ad4	Adeno4.U3	CAAGGACTACCAGGCCGTCA	Ad4 hexon
		Adeno4.L1	TTAGCATAGAGCATGTTCTGGC	
F		AdF1	ACTTAATGCTGACACGGGCAC	Long fibre gene
	Ad40	K402	CAC TTA ATG CTG ACA CG	
	Ad41	K403	ACT GGA TAG AGC TAG CG	

Source: [27, 28].

**Table 2:** ATTC viral control strains.

Virus	Reference number	Strain
Human adenovirus 40	ATCC VR-931	Strain Dugan
Human adenovirus 41	ATCC VR-930	Strain Tak (73-3544)
Human adenovirus 2	ATCC VR-846	Strain Adenoid 6
Human adenovirus 6	ATCC VR-6	Strain Tonsil 99
Human adenovirus 7	ATCC VR-7	Strain Gomen
Human adenovirus 3	ATCC VR-3	Strain GB
Human adenovirus 1	ATCC VR-1	Strain Adenoid 71
Adenovirus T 21	ATCC(R) VR-256	Strain AV 1645
Human adenovirus 4	ATCC VR-1572	Strain R1-67
Adenovirus 5	ATCC VR-1516	
